# Effect of quantum iterative reconstruction on coronary stent assessment in ultra-high resolution photon-counting detector CT: a phantom study

**DOI:** 10.1186/s41747-026-00739-6

**Published:** 2026-05-20

**Authors:** Søren Thorup Scheuer, Martin Weber Kusk, Henrik Sæderup, Lotte Grumsen, Alexandru Diaconu, Hans Pauli Arnoldson, Lisette Okkels Jensen, Michael Boelstoft Holte, Niels Peter Rønnow Sand, Kristian Tækker Madsen

**Affiliations:** 1 Department of Cardiology, University Hospital of Southern Denmark, Esbjerg, Denmark; 2 Department of Radiology, University Hospital of Southern Denmark, Esbjerg, Denmark; 3 Department of Maxillofacial Surgery, University Hospital of Southern Denmark, Esbjerg, Denmark; 4 Department of Cardiology, Odense University Hospital, Odense, Denmark

**Keywords:** Artifacts, Computed tomography angiography, Coronary arteries, Phantoms (imaging), Stents

## Abstract

**Objective:**

This study seeks to assess the impact of quantum iterative reconstruction (QIR) on objective measures of contrast-to-noise ratio (CNR), lumen diameter, and stent strut thickness using photon-counting detector computed tomography (PCD-CT) in ultra-high resolution mode (UHR) in a coronary phantom.

**Materials and methods:**

A three-dimensional printed phantom (four parallel vessels; 3 vessels with varying 3.5-mm stent types) was applied. Demineralized water, mixed with iodinated contrast, was circulated through the phantom by a dynamic pump. Images were acquired on a commercially available PCD-CT system using retrospective helical acquisition in UHR mode at both 120 and 140 kVp. Images were reconstructed at four QIR-levels (1‒4) using three sharp vascular kernels (Bv56u, Bv68u, and Bv76u). Quantitative evaluations of lumen diameter (mm) and stent strut thickness (mm) were assessed by attenuation profiles using an in-house MATLAB script.

**Results:**

Increasing QIR level from 1 to 4 improved CNR from 1.5 (95% confidence interval [CI] 1.31‒1.70) (QIR-level 1) to 3.33 (95% CI: 2.89‒3.77) (QIR-level 4), *p* < 0.001. There was no change (QIR-level 1 to 4) in lumen diameter, range 2.95 mm (95% CI: 2.89‒3.02) to 2.97 mm (95% CI: 2.91‒3.03) (*p* = 0.890) or strut thickness, range 0.62 mm (95% CI: 0.59‒0.65) to 0.61 mm (95% CI: 0.58‒0.65 ((*p* = 0.870), regardless of contrast concentration or reconstruction kernel.

**Conclusion:**

Increasing QIR-level in UHR mode PCD-CT in a stented coronary phantom improved CNR without affecting objective measures of lumen diameter or stent strut thickness under controlled phantom conditions.

**Relevance statement:**

There has been debate regarding the negative impact of iterative reconstruction on spatial resolution, potentially increasing blooming artifacts caused by coronary stents, which is of special concern when imaging small stent lumens. Currently, no studies have assessed the effect of QIR on the objective assessment of coronary stents.

**Key Points:**

- Some earlier implementations of iterative reconstruction negatively impacted spatial resolution, raising concerns for small-vessel and stent imaging.
- No prior studies examined the effect of QIR on coronary stent assessment.
- The effect of QIR was evaluated at four levels (1‒4) in a stented coronary phantom.
- Increasing QIR to the highest level significantly improved luminal CNR.
- Objective measures of stent dimensions remained unchanged across all QIR levels.

**Graphical Abstract:**

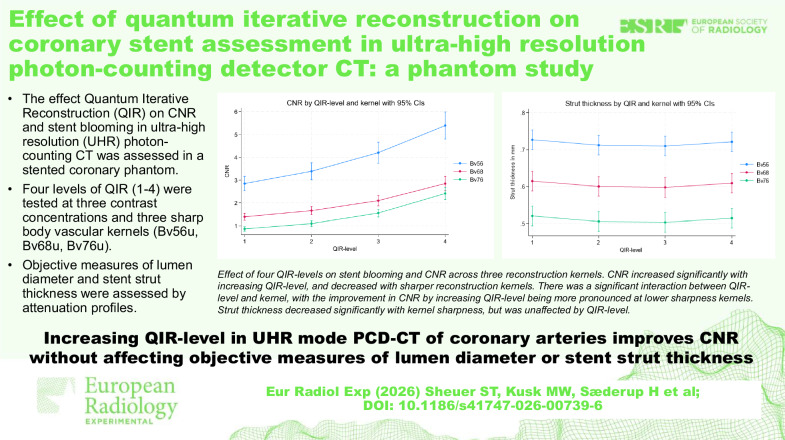

## Background

Current American Heart Association guidelines recommend limiting routine use of coronary computed tomography angiography (CTA) to assessment of coronary stents ≥ 3 mm in diameter due to partial volume and beam-hardening artifacts negatively affecting diagnostic confidence for evaluating small caliber stents [1]. With recent advancements in scanner technology by photon-counting detector computed tomography (PCD-CT), spatial resolution has been markedly improved compared to conventional energy-integrating detector CT scanners (150‒200 µm *versus* 400‒450 µm, respectively) [2, 3]. PCD-CT allows for reconstruction in both standard-resolution mode with spectral capabilities (slice thickness 0.4 mm) and ultra-high resolution (UHR) mode with increased spatial resolution (slice thickness 0.2 mm) at the expense of spectral information. For coronary stent assessment, UHR mode has been shown to provide improved lumen visualization compared to standard-resolution with spectral reconstructions [4]. In the context of coronary CTA, UHR acquisition translates to improved visualization of vessel lumen in the presence of calcifications and metallic stents [5, 6], primarily by reducing blooming artifacts attributable to partial volume effects [7].

However, improved spatial resolution and high sharpness kernels leads to higher image noise [8]. Iterative reconstruction algorithms can improve the signal-to-noise ratio in CTA, but there has been concerns regarding a negative impact on spatial resolution [9], which is undesirable when imaging small stent lumens due to a potential increase in stent blooming. The PCD-CT system (NAETOM Alpha, Siemens Healthineers), employs “Quantum Iterative Reconstruction” (QIR) with levels ranging from 1 to 4. QIR is a model-based iterative reconstruction algorithm for PCD-CT tailored to handle the increased complexity of energy-resolved spectral data. It has previously been demonstrated that QIR level 4 provides the optimal image sharpness for non-stented CTA images [6], however, this evaluation was performed using medium-smooth resolution vascular kernels and virtual monoenergetic reconstructions of standard-resolution PCD-CT, not optimized for stent visualization. Similarly, the effect of increasing QIR-level was tested in abdominal imaging with virtual monoenergetic imaging reconstructed at 2 mm with the Br36 kernel, showing improved image quality and reduced noise without a negative impact on image texture or attenuation values [10]. One study evaluated the effect of QIR-level in low-dose UHR imaging of lung parenchyma, and found a slight decrease in spatial resolution with increasing QIR-level [11]. The authors reported QIR-level 3 to provide optimal subjective image sharpness when reconstructing images at a high sharpness kernel (Bl64).

No previous studies have evaluated the effect of reconstruction at different QIR-levels in UHR PCD-CT for assessment of high-attenuation coronary stents. Therefore, the aim of the present study was to explore whether different QIR levels affected objective measures of contrast-to-noise ratio (CNR), lumen diameters and stent strut thickness in UHR scan mode at varying reconstruction kernels, contrast concentrations, and tube voltages.

## Methods

### Ethical considerations

Ethical approval and informed consent were not required for this study, as it was a phantom experiment and did not involve human participants or patient data.

### Phantom and experimental setup

**Fig. 1:**
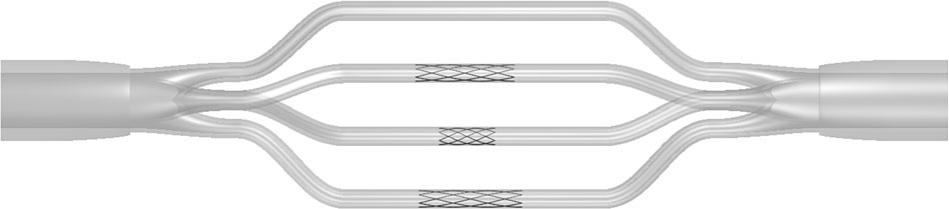
Three dimensional-printed vessel phantom. Computer-generated image of the 3D-printed phantom containing four parallel vessels, each with an internal diameter of 3.5 mm and a wall thickness of 1 mm. 3D, Three-dimensional

A three-dimensional printed vessel phantom containing four parallel vessels with an internal diameter of 3.5 mm and a wall thickness of 1 mm was prepared (Fig. 1). Three commercially available coronary stents (Evermine50, Meril Life Sciences Pvt. Ltd., Vapi, India; Xience Pro, Abbott Vascular; and Synergy, Boston Scientific) with a diameter of 3.5 mm were deployed in independent vessels with one vessel being left non-stented serving as a reference vessel. Stent characteristics are summarized in Table 1.

**Table 1:** Characteristics of the coronary stents deployed in the vessel phantom

Model	Manufacturer	Diameter at 16 atm (mm)	Length (mm)	Strut thickness (µm)	Material	Drug eluting
Evermine50	Meril Life Sciences	3.5/3.81	13	50	Cobalt- chromium	Yes
Xience Pro	Abbott Vascular	3.5/3.70	23	81	Cobalt-chromium	Yes
Synergy	Boston Scientific	3.5/3.79	24	79	Platinum-chromium	Yes

These three coronary stents (3.5 mm nominal diameter) from different manufacturers were expanded at 16 atm in the three-dimensional-printed phantom. Two stents were made of cobalt-chromium alloy and one of platinum-chromium. The fourth, empty phantom vessel served as a reference vessel

The phantom was connected to a pump capable of mimicking physiologic conditions through adjustable heartrate and stroke volume (SuperPump, ViVitro Labs). The pump was set at a frequency of 60 beats per min and a stroke volume of 75 mL, resulting in a cardiac output of 4.5 L/min. Slight movement along the *x* and *y* axes was introduced by the pulsatile flow generated by the pump, mimicking coronary movement. The pump circulated demineralized water deprived of air bubbles, mixed with 350 mg/mL iodinated contrast (Omnipaque, GE Healthcare). Contrast concentrations were adjusted by stepwise dilution to achieve target luminal attenuations of approximately 300, 400 and 500 HU (contrast concentrations 1, 2, and 3), as verified on reconstructed images. Three contrast concentrations were included to assess potential interactions between contrast attenuation and the effect of QIR-level. To achieve realistic attenuation properties, the phantom was placed in a cylindrical tank (diameter 32 cm) filled with water. The tank was placed with the phantom at the isocenter of the scanner.

### CT acquisition and reconstruction parameters

All images were acquired on a first-generation dual-source PCD-CT system (NAEOTOM Alpha, Siemens Healtineers), software version VA50. Image data were acquired by retrospectively electrocardiographically-gated helical, UHR mode (collimation 120 × 0.2 mm), using an onboard synthetic electrocardiogram-generator to provide gating signal at 60 beats. Tube current was fixed at 99 mAs for all acquisitions. For each of the three concentrations of contrast media, three repeated scans were performed to account for variations in movement introduced by the pulsatile flow in the vessel phantom. Acquisitions were performed at both 120 and 140 kVp, for a total of 18 image datasets. Axial images were reconstructed in the 75% phase with a slice thickness of 0.2 mm and a reconstruction increment of 0.15 mm. Images were reconstructed using a 768 × 768 matrix and a reconstruction field of view of 160 mm, resulting in an in-plane pixel size of 0.21 × 0.21 mm^2^, and thus near-isotropic resolution. No additional *z*-filtering or postprocessing beyond standard reconstruction and QIR was applied. The 768 × 768 matrix was selected to enable small in-plane pixel spacing and to fully exploit the increased spatial resolution afforded by UHR-acquisition.

Reconstructions were made using three sharp body-vascular kernels (Bv56u, Bv68u, and Bv76u). For each kernel, images were reconstructed at QIR levels 1–4 to span the full clinically available range of reconstruction strengths. QIR-level 0 (*i.e.*, QIR disabled) was not included, as UHR imaging without iterative reconstruction is unlikely to be used clinically due to excessive noise. Three sharp body vascular kernels were chosen (Bv56u, Bv68u, and Bv76u) as these kernels represent the range most commonly applied for coronary stent imaging. All acquisition and reconstruction parameters are listed in Table 2.

**Table 2:** Acquisition and reconstruction parameters

Rotation time (ms)	250
Temporal resolution (ms)	66
Collimation (mm)	24
Acquisition mode	Retrospectively gated helical
Pixel size (mm)	0.21 × 0.21
Helical pitch	0.20–0.25
Tube voltage (kVp)	120/140
Tube current (mAs)	99 (fixed)
Slice thickness (mm)	0.2
Slice increment (mm)	0.15
Image matrix	768 × 768
Kernel	Bv56u, Bv68u, Bv76u
Iterative reconstruction	QIR 1, 2, 3, and 4

### Quantitative analysis

**Fig. 2:**
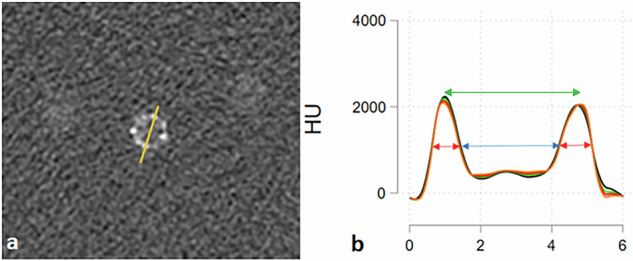
Measurement of stent attenuation profiles. **a** Cross-sectional stent image with a measurement line drawn through two opposing struts. **b** Corresponding attenuation profile along the line, generated using an in-house MATLAB script. Lumen diameter was defined as the inner distance at FWHM of lumen attenuation (blue arrow), and strut thickness as the average width at FWHM of the two strut peaks (red arrows). Stent diameter was defined by the distance between the two attenuation peaks. FWHM, Full width at half maximum

For quantitative evaluations of stent dimensions and CNR an in-house MATLAB (MathWorks Inc.) script was used. For each stent, attenuation intensity profiles were created by drawing a line through two opposing stent struts in a cross section of each stent (Fig. 2). Optimal placement was aided by an interactive plot to ensure crossing at maximum intensity values. The profile line was drawn across the same stent struts in all scans and automatically copied to the same exact location across all reconstructions per acquisition, thus eliminating reader-induced variability. One hundred sampled attenuation values were measured for each line, using bicubic interpolation, and physical distances calculated from spatial reference information embedded in the Digital Imaging and Communications in Medicine‒DICOM metadata.

Inner stent diameter (lumen diameter) was defined as the inner distance between the stent attenuation profile at full width at half maximum, of lumen HU. The term stent diameter refers to the outer stent diameter, defined as the distance from the peak HU-values from each opposing stent strut. Strut thickness was defined as the average of the two HU-profile widths at full width at half maximum.

Image noise was measured in the phantom by placing three region of interest (ROI) with an area of 300 mm^2^ in the water tank and was defined as the average standard deviation of attenuation in the ROIs. Circular ROIs were drawn in the lumen of the stented segments in the phantom vessels on all performed scans, and the size, shape and position of ROIs and attenuation line were automatically kept constant between the series. CNR was calculated as:

$${CNR}=\frac{{\mathrm{Average}}\,{\mathrm{signal}}\,{\mathrm{ROI}}-{\mathrm{Average}}\,{\mathrm{background}}\,{\mathrm{ROI}}}{{\mathrm{Standard}}\,{\mathrm{deviation}}\,({\mathrm{background}}\,{\mathrm{ROI}})}$$

### Statistical analysis

Continuous outcome variables were described using means and compared using Wald test. Normality of distribution for each outcome was assessed using visual inspection of histograms and Q–Q plots. A log transformation was applied to CNR values to meet the assumptions of normality required, and model estimates were exponentiated to obtain geometric means and corresponding 95% confidence intervals (CI). A linear mixed-effects regression model was employed to evaluate the effect of QIR on each outcome variable (CNR, lumen diameter, strut thickness), with QIR, reconstruction kernel, contrast concentration, and tube voltage treated as a fixed effects. A random intercept was included to account for repeated measurements within scan repeats.

Interaction terms between QIR and covariates were added individually to assess potential effect modification. Given the exploratory nature of the study and the strong a priori hypotheses regarding the direction of QIR effects, no formal correction for multiple comparisons was applied. Results were interpreted with emphasis on effect sizes and consistency across reconstruction settings. A two-tailed *p*-value < 0.05 was considered statistically significant. All statistical analyses were conducted using STATA 18BE (StataCorp).

## Results

A total of 216 reconstructions were analyzed across three stent types, three contrast concentrations, two tube voltages (120 and 140 kVp), three reconstruction kernels, and four QIR levels (1–4).

### Image noise and attenuation

Mean image noise in the background water phantom was 10.99 ± 0.60 HU (mean ± standard deviation) across all scans. Mean luminal HU for the three contrast concentrations were 339 ± 5, 389 ± 5, and 547 ± 4, respectively.

### CNR

**Fig. 3:**
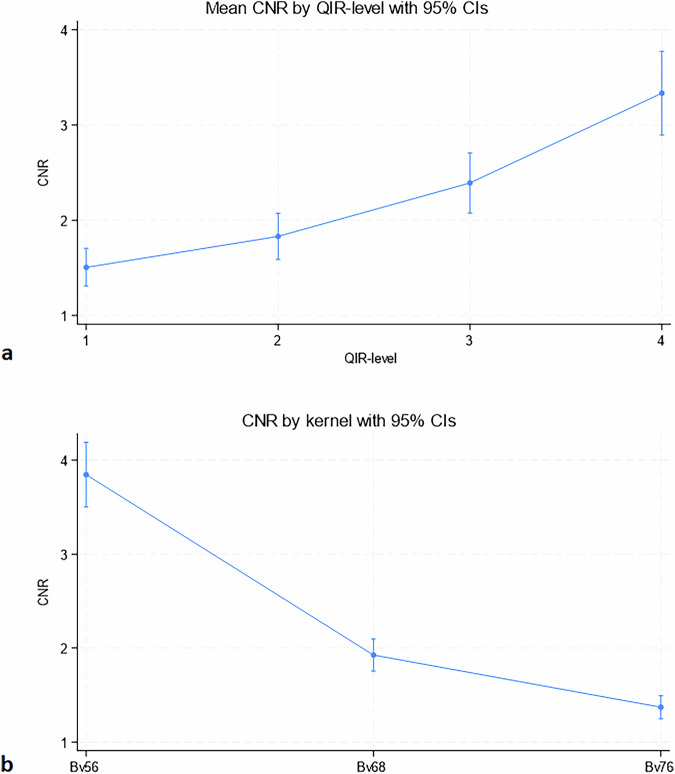
Effect of reconstruction settins on CNR. Measurements represent mean measurements across all three stents. **a** CNR increased progressively with higher QIR levels, from 1.5 (95% CI: 1.31–1.70) at QIR-1 to 3.33 (95% CI: 2.89–3.77) at QIR-4 (*p*-value for trend < 0.001). **b** CNR decreased with sharper kernels, from 3.85 (95% CI: 3.50–4.19) at Bv56u to 1.37 (95% CI: 1.25–1.49) at Bv76u. CNR, Contrast-to-noise ratio; QIR, Quantum iterative reconstruction

CNR increased with increasing QIR level from 1.5 (95% CI: 1.31–1.70) to 3.33 (95% CI: 2.89–3.77) for QIR-level 1 and 4, respectively, *p* < 0.001 (Fig. 3a). CNR decreased with higher sharpness kernels (Bv56u, Bv68u and Bv76u): 3.85 (95% CI: 3.50–4.19), 1.93 (95% CI: 1.75–2.10) and 1.37 (95% CI: 1.25–1.49), respectively, *p* < 0.001 (Fig. 3b).

**Fig. 4:**
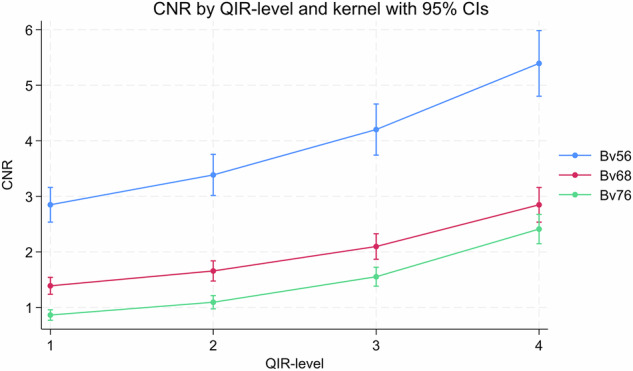
Interaction between QIR-level and reconstruction kernel on CNR. CNR plotted across QIR levels 1–4 for kernels Bv56u, Bv68u, and Bv76u. A significant interaction was observed (*p* = 0.007), with the CNR difference between Bv56u and Bv76u being greater at QIR-4 than at QIR-1 (-2.98 *versus* -1.99). CNR, Contrast-to-noise ratio; QIR, Quantum iterative reconstruction

**Fig. 5:**
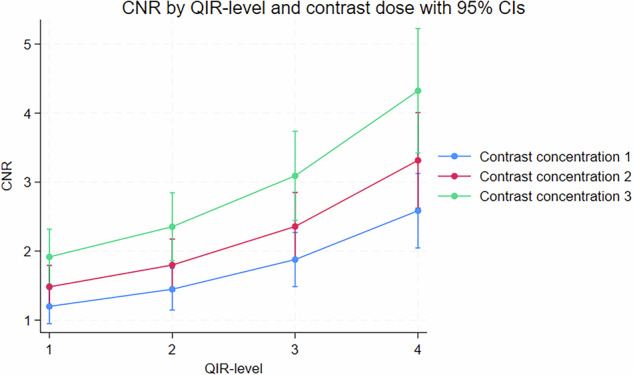
Interaction between QIR-level and contrast concentration on CNR. CNR plotted across QIR levels 1–4 for three contrast concentrations. The positive effect of QIR level 4 *versus* 1 did not change significantly with increasing contrast concentration, from 1.38 (95% CI: 0.79–1.98) at contrast concentration 1 to 2.40 (95% CI: 1.42–3.39) at contrast concentration 3 (*p*-value for interaction = 0.083). CNR, Contrast-to-noise ratio; QIR, Quantum iterative reconstruction

There was a significant interaction between QIR-level and kernel, with the increase in CNR by employing QIR-level 4 *versus* 1 being more pronounced at lower sharpness kernels: +2.98 (95% CI + 2.34; + 3.63) and +1.99 (95% CI + 1.66; + 2.31), for kernels Bv56u and Bv76u, respectively, *p* = 0.007 (Fig. 4). The effect of QIR-level 4 *versus* 1 on CNR did not change significantly with increasing contrast concentration (+ 1.38 [95% CI: 0.79–1.98] and +2.40 [95% CI: 1.42–3.39], *p* = 0.083 at contrast concentrations 1 and 3, respectively) indicating no interaction between QIR strength and contrast attenuation (Fig. 5). There was no impact of tube voltage on CNR when pooling across all QIR levels (2.60 [95% CI: 2.32–2.87] and 2.56 [95% CI: 2.26–2.85], *p* = 0.846), for 120 and 140 kVp, respectively.

### Quantitative measurement of strut thickness and lumen diameter

**Fig. 6:**
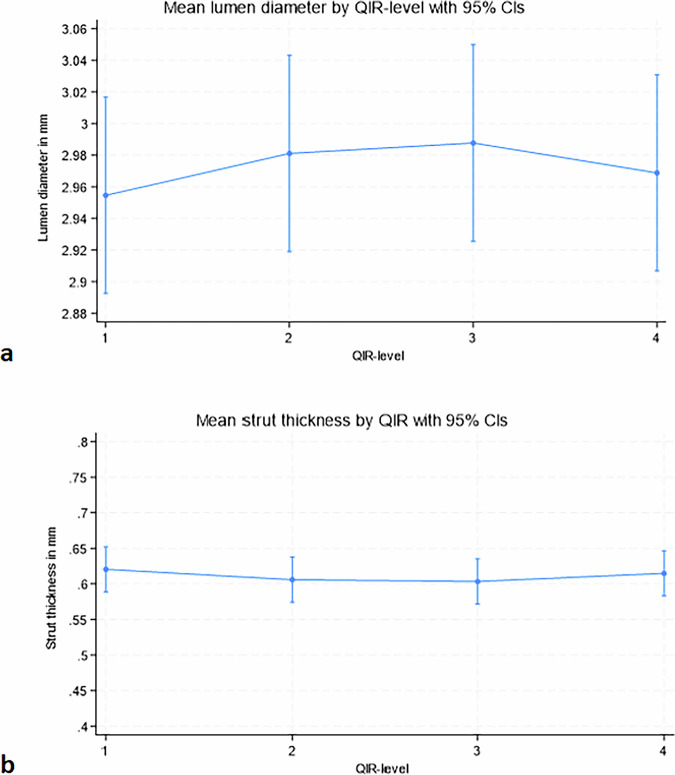
Effect of QIR-level on stent dimensions. Measurements are shown pooled across stents to illustrate relative reconstruction-dependent trends; individual stent sizes differed slightly but demonstrated consistent behavior across QIR levels and kernels. **a** Strut thickness and (**b**) lumen diameter plotted across QIR levels 1–4. No significant differences were observed: lumen diameter ranged from 2.95 mm (95% CI: 2.89–3.02) to 2.97 mm (95% CI: 2.91–3.03) (*p* = 0.890), and strut thickness from 0.62 mm (95% CI: 0.59–0.65) to 0.61 mm (95% CI: 0.58–0.65) (*p* = 0.870). QIR, Quantum iterative reconstruction

**Fig. 7:**
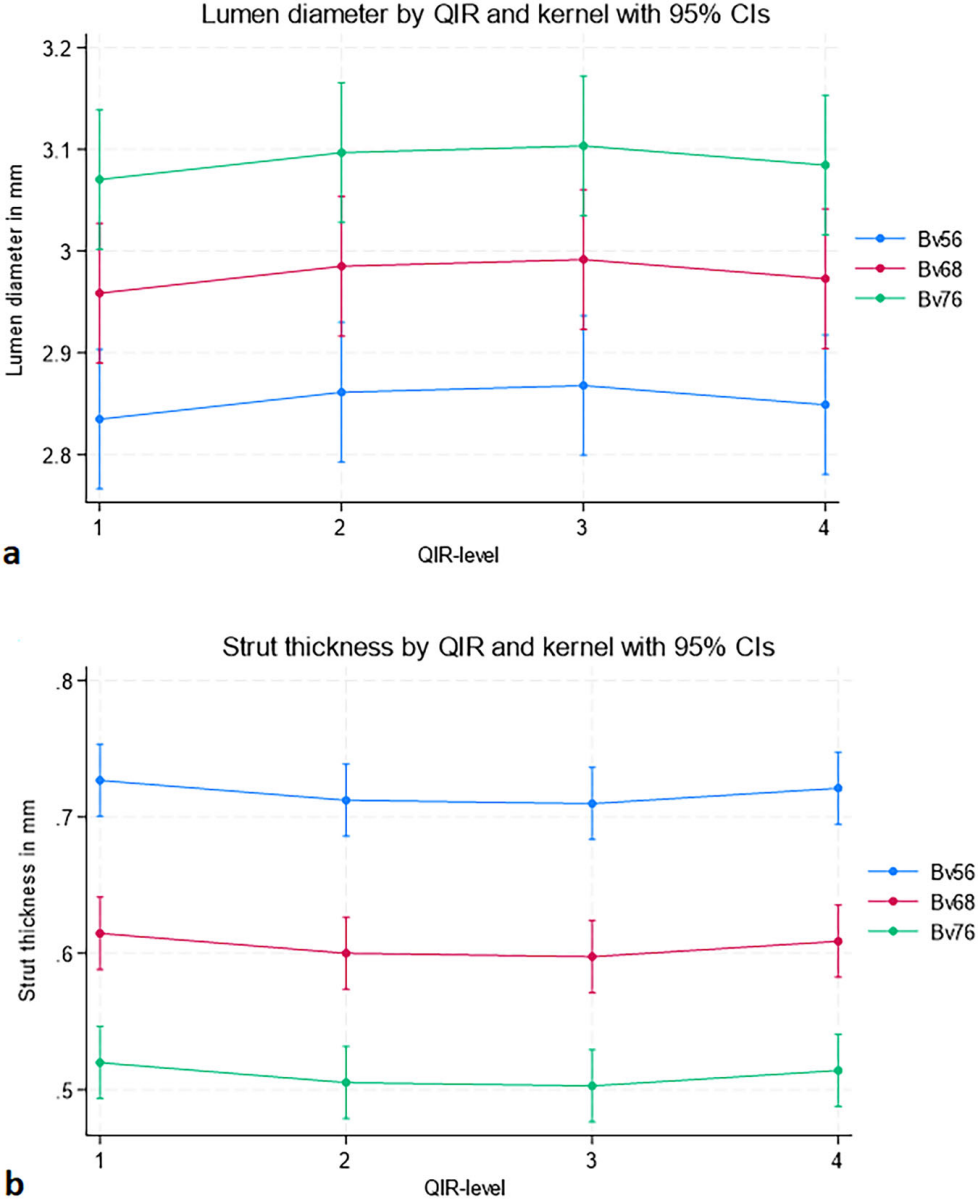
Effect of reconstruction kernel on stent dimensions across QIR levels. **a** Lumen diameter increased significantly with sharper kernels, from 2.83 mm (95% CI: 2.77–2.90) at Bv56u to 3.07 mm (95% CI: 3.00–3.14) at Bv76u (*p* < 0.001). **b** Strut thickness decreased significantly with kernel sharpness, from 0.73 mm (95% CI: 0.70–0.75) at Bv56u to 0.52 mm (95% CI: 0.49–0.55) at Bv76u (*p* < 0.001). No significant interaction with QIR-level was observed. Abb, QIR, Quantum iterative reconstruction

Lumen diameter and strut size did not differ significantly across QIR levels. Lumen diameter ranged from 2.95 mm (95% CI: 2.89–3.02 mm) to 2.97 mm (95% CI: 2.91–3.03 mm), *p* = 0.890 (Fig. 6a), and strut thickness from 0.62 mm (95% CI: 0.59–0.65 mm) to 0.61 mm (95% CI: 0.58–0.65 mm), *p* = 0.870 (Fig. 6b). Higher sharpness kernels (Bv76u *versus* Bv56u) increased the luminal diameter (mean 3.07 mm [95% CI: 3.00‒3.14] *versus* 2.83 [95% CI: 2.77*‒*2.90], *p* < 0.001) and decreased strut thickness (mean 0.52 mm [95% CI: 0.49*‒*0.55] *versus* 0.73 [95% CI: 0.70*‒*0.75], *p* < 0.001), respectively (Fig. 7). There was no interaction between QIR-level and kernel, contrast concentration or kVp for measures of lumen diameter or strut thickness (Fig. 5). Similarly, no significant differences were observed across kVp levels: mean luminal diameter was 2.96 mm (95% CI: 2.92–3.01) at 120 kVp *versus* 2.98 mm (95% CI: 2.94–3.03) at 140 kVp (*p* = 0.540), and mean strut thickness was 0.62 mm (95% CI: 0.60–0.64) at 120 kVp *versus* 0.60 mm (95% CI: 0.58–0.63) at 140 kVp (*p* = 0.408). Measured stent strut thickness values were substantially larger than manufacturer-specified nominal strut thicknesses (50–80 µm) across all reconstruction settings, consistent with expected blooming effects. Compared with *in vitro* reference values, CT-based measurements showed a mean underestimation of the outer stent diameter of 2.3%. At the sharpest reconstruction kernel (Bv76u), the mean visible lumen diameter corresponded to 82.0% of the *in vitro* reference outer stent diameter.

## Discussion

This phantom study evaluated the impact of varying levels of QIR on CNR and quantitative assessment of coronary stent and lumen dimensions, using UHR PCD-CT angiography across three high sharpness kernels, three contrast concentrations, and two tube voltages. It was observed that increasing QIR levels significantly improved CNR without compromising relative measurements of stent strut thickness or lumen diameter, whilst sharper reconstruction kernels substantially influenced stent visualization by reducing measures of stent strut thickness and increasing visible luminal diameter. Trends in apparent stent diameter were consistent across the three investigated stents, supporting pooled analysis of reconstruction-dependent effects.

In the present study, we selected the UHR mode to evaluate the impact of different QIR levels, as the thinner slice thicknesses by the UHR mode are associated with markedly increased image noise. Similarly, we included only high-sharpness reconstruction kernels, known to be most effective for stent assessment [8, 12], despite their tendency to amplify image noise at higher levels.

Our results are in line with a previous study by Vecsey-Nagy et al, demonstrating that increasing QIR in UHR PCD-CT does not affect objective or subjective image sharpness, but reduces image noise, while stenosis measurements are unaffected in non-stented patients [13]. A similar conclusion has been made in PCD-CT angiographies of the lower leg [14].

Our results align with previous work demonstrating that sharper kernels improve stent lumen visualization by reducing blooming artifacts, as seen by a significant reduction in measured strut thickness and increased lumen diameter at higher sharpness kernels [8, 12, 15]. However, the differences demonstrated are in the order of one to two pixels, the clinical relevance of which will have to be weighed against CNR reduction in higher kernels. Preferably, image reading should be performed using different kernels to leverage both high spatial and contrast resolution. Based on the present findings, an intermediate-to-high sharpness reconstruction kernel such as Bv68u appears to provide the balanced trade-off for ultra-high-resolution coronary stent imaging.

In a retrospective *in vivo* study, Geering et al evaluated the effect of various reconstruction parameters on stent visualization, and found the best overall subjective image quality at 0.2 mm/Bv72 reconstructions [15]. Of note, they find a 20‒30% underestimation of the inner stent lumen compared to in-vitro conditions when reconstructing at Bv72 and Bv56, which is comparable to our findings in the current phantom setup.

Automated tube voltage selection works by selecting the combination of tube voltage and current that produces the prescribed image quality at the lowest radiation dose, potentially reducing the risk of stochastic damage associated with CT. The fact that lumen diameters and CNR were unaffected by the tube voltages investigated (120 and 140 kVp), suggests that automated tube voltage selection may be used in UHR-scanning of stents within the limited kVp-range allowed for the UHR scanmode, with no concerns regarding measurement accuracy, although extrapolation to in-vivo conditions should be made with caution. Due to the very high attenuation of strut material, this also supports the hypothesis that blooming artifacts are, primarily, caused by partial volume effects [7], rather than beam hardening. Definitive conclusions regarding underlying mechanisms for stent blooming are, however, beyond the scope of this phantom study, and should be investigated in a separate study.

Some important limitations of this study should be emphasized. First, only 3.5-mm stents were evaluated. Although reconstruction-related blooming effects are expected to be independent of nominal stent diameter for a given stent design, the relative impact on visible lumen area will be greater in smaller stents. Therefore, extrapolation of the present findings to smaller stent diameters should be made with caution. Of note, a recent prospective clinical study of 79 stented patients referred for ICA, sensitivity and specificity for identifying in-stent restenosis > 50% was 95% and 84%, respectively [16]. A subgroup analysis did not identify significant differences in sensitivity or specificity based on stent size. Second, as nominal coronary stent strut thicknesses (50‒80 µm) are substantially below the spatial resolution of clinical PCD-CT (200 µm), absolute validation of strut dimensions is not feasible without a physical ground truth (*e.g*., micro-CT), and our findings therefore reflect relative reconstruction-dependent differences. Third, given the nature of this phantom study, factors inherent to *in vivo* coronary imaging, including cardiac motion, vessel tortuosity, calcified plaque, and heterogeneous surrounding tissues, were not represented in the present model and may influence both image quality and apparent stent dimensions. For a complete and rigorous characterization of spatial resolution metrics, line-pair measurements, edge-based modulation transfer function, or more appropriately, task transfer function [17] could have been employed, but would require a different phantom setup outside the scope of our study. Furthermore, our measurement method, although simplistic, provides a clinically relevant and easy-to-interpret result for reading radiologists. Full width at half maximum‒FWHM provides an indirect quantification of blooming artifacts, having been used for that purpose in simulated coronary calcifications [18]. However, the inclusion of full-width, tenth-maximum measurements could have been informative in the context of accuracy for the detection of neointimal hyperplasia with relatively low density.

In the present study, only a single maximum allowable tube current was used. This highlights one of the challenges of using UHR, namely that the available tube effect is halved and scan times are increased compared to standard-mode PCD-CT. This also accounts for the relatively low CNR values achieved. The rule of thumb that CNR should be at least five, for reliable object detection [19], suggests that vessel to background tissue delineation could be impaired at the settings used in our study. Notably, a CNR above five was only achievable when applying QIR-level 4 to images reconstructed at Bv56u. Other studies have suggested using UHR mode for acquisition [20], but reconstruct at standard resolution (0.4 mm) to recover CNR, while taking advantage of the increase in resolution conferred by the small focal spot. This was, however, beyond the scope of our investigation. Most importantly, phantom studies are, by their nature, of limited generalizability, and our results should be tested in an in vivo setting. Such a study can also shed light on the effect of QIR and kernels on visibility and quantification of in-stent lesions.

In addition, we only performed a quantitative analysis, which did not measure reader confidence in the assessment of stent lumens; however, CNR is often used as a surrogate marker of image quality. As suggested by the Rose model [19], improving CNR increases the sensitivity of the human eye. Likewise, noise structure measurements (*e.g*., noise-power spectra) were not analyzed. Optimizing image quality may not only apply to the assessment of stents, but also to the evaluation of the degree of stenosis and high-risk plaque features in non-stented patients.

In conclusion, our results suggest that increasing QIR-level in UHR mode PCD-CT of coronary arteries increases CNR without affecting objective measures of lumen diameter or stent strut thickness under the investigated phantom conditions.

## Data Availability

The datasets used and/or analyzed during the current study are available from the corresponding author on reasonable request.

## Abbreviations

CI: Confidence interval
CNR: Contrast-to-noise ratio
CT: Computed tomography
CTA: Computed tomography angiography
PCD-CT: Photon-counting detector computed tomography
QIR: Quantum iterative reconstruction
ROI: Region of interest
UHR: Ultra-high resolution
